# Sex and race influence objective and self-report sleep and circadian measures in emerging adults independently of risk for bipolar spectrum disorder

**DOI:** 10.1038/s41598-020-70750-3

**Published:** 2020-08-13

**Authors:** Madison K. Titone, Brae Anne McArthur, Tommy H. Ng, Taylor A. Burke, Laura E. McLaughlin, Laura E. MacMullen, Namni Goel, Lauren B. Alloy

**Affiliations:** 1 Department of Psychology, Temple University, 1701 N 13th St, Philadelphia, PA USA; 2 Department of Psychology, University of Calgary, Calgary, AB Canada; 3 Department of Psychiatry and Human Behavior, Alpert Medical School Brown University, Providence, RI USA; 4 Department of Psychiatry, Perelman School of Medicine, University of Pennsylvania, Philadelphia, PA USA; 5 Department of Psychiatry and Behavioral Sciences, Rush University Medical Center, Chicago, IL USA

**Keywords:** Psychology, Risk factors

## Abstract

There is a need to better understand key factors that impact sleep and circadian function for young adults of differing races and sexes. Sex and race are common factors contributing to disparities in health outcomes; however, the influence of these variables on sleep and circadian patterns for young adults are not well known. Multiple objective and self-report facets of sleep and circadian function were assessed (melatonin onset, actigraphy, and sleep diaries) in an ecological momentary assessment study of 150 emerging adults (*M*_age_ = 21.8 years; 58.7% female; 56% White, 22.7% Black, 21.3% Other ethnicity) at high or low risk for bipolar spectrum disorder (BSD). Controlling for BSD risk status, sex and race were significant predictors of objective and self-reported sleep and circadian rhythm measures. Males self-reported better sleep efficiency and exhibited later dim light melatonin onset phase than females, whereas females exhibited more actigraphy-measured sleep periods. White participants exhibited more actigraphy-measured total sleep time (TST), better sleep efficiency, and fewer sleep periods, and more self-reported TST and better sleep efficiency than Black participants. Our findings enhance the literature by utilizing robust measurement of sleep and circadian parameters to extend previous findings to a young adult sample at high or low risk for BSD.

## Introduction

Although sleep is a critical component of a healthy lifestyle^1,2^, many young adults do not obtain enough quality sleep^3,4^. Optimal sleep and circadian function are imperative for cognitive functioning (memory, mood regulation, etc.), metabolism, appetite regulation, immune and hormone functioning, and cardiovascular health^2,5^. Moreover, sleep and circadian disturbances relate to hypertension, heart disease, diabetes, obesity, and mortality^2,5^, and play a key role in the onset and course of Bipolar Spectrum Disorder (BSD)^6^.

In adult samples, females objectively slept better than males in the laboratory with earlier timing, longer duration of sleep, shorter sleep onset latency, better sleep efficiency and more slow-wave sleep^7,8^. Similarly, in a young adult sample, dim light melatonin onset (DLMO) phase was earlier in females than in males^9^, and in an adolescent sample, based on actigraphic estimates, wake after sleep onset (WASO) was greater and total sleep time was reduced for boys compared to girls^10^. However, females report significantly more sleep problems than males^11^ and less high-quality, uninterrupted sleep^12,13^. Further, beyond sleep measures, the intrinsic circadian period has been shown to be significantly shorter in healthy adult females than males in some studies^14,15^, and females also have been reported to have an earlier entrained melatonin circadian phase than males^16^.

Objective sleep studies have found decreases in slow-wave sleep (SWS) and increases in stage 1 and 2 non-rapid eye movement (NREM) sleep in Blacks compared to Whites^17–21^. Sleep duration also has been reported to be shorter, and sleep efficiency and self-rated sleep quality found to be poorer in Blacks than Whites^17,22–25^. Population-based studies also have reported racial differences using self-reported sleep^26–28^ or wrist actigraphy^29^, whereby Blacks are more likely to sleep less than Whites. The race-sleep relationship persists independently of socioeconomic status^17^, and there is an established association of self-identified race with sleep health inequalities^30–32^. In addition, beyond sleep, a number of studies show Blacks also have a shorter endogenous circadian period, larger phase advances, and smaller phase delays than Whites^15,32–36^; by contrast, self-reported chronotype as measured by the Morningness-Eveningness Questionnaire (MEQ)^37^ did not significantly differ between Blacks and Whites^32^.

There are several limitations of the existing literature. Sleep in some of these studies was assessed solely via self-report and/or along a single dimension or item, and research has shown that individuals overestimate self-reported sleep when compared to objective measures^10,38^. Furthermore, research including objective measures of sleep and circadian rhythms often derives from laboratory studies, which utilize well-controlled environments, but reduce generalizability. Much less is known about objective sleep and circadian rhythm parameters in emerging adults’ daily lives. Additionally, most studies have been cross-sectional, and few have investigated sex and racial differences in emerging adults at high or low risk for BSD.

To address these limitations, we examined multiple facets of sleep and circadian rhythms including objective and self-report assessments in a naturalistic ecological momentary assessment (EMA) study using a large sample of emerging adults, some of whom were at risk for BSD.

Accordingly, the aims and hypotheses of this study were as follows: 1. To examine the influence of sex and race on sleep and circadian measures in a novel population and setting. Given the research outlined above indicating poorer self-reported, but better objective, sleep quality in females, we hypothesized females would report poorer sleep ratings, but would show better objective sleep outcomes, compared to males. Because previous literature demonstrates poorer objective and subjective sleep quality in Black compared to White participants, we also hypothesized White participants would show better self-reported and objective sleep indices compared to Black participants. 2. To strengthen the rigor of construct measurement for sleep and circadian rhythms in a naturalistic EMA study by examining multiple facets of sleep and circadian rhythms including objective and self-report assessments. We hypothesized self-report and objective sleep indices would be significantly different from each other in a naturalistic EMA study.

## Results

### Relationships between sleep and circadian measures

Bivariate Pearson correlations between the primary sleep and circadian measures indicated that actigraphy-measured total sleep time (TST) was positively associated with actigraphy-measured sleep efficiency (SE) (*r*(129) = 0.28, *p* = 0.001), actigraphy-measured number of sleep periods (SP) (*r*(129) = 0.26, *p* = 0.003), and average self-reported TST (*r*(129) = 0.43, *p* < 0.001). Actigraphy-measured SE was positively associated with self-reported SE (*r*(127) = 0.19, *p* = 0.035). Actigraphy-measured number of SP was negatively associated with average self-reported TST (*r*(129) = − 0.18, *p* = 0.045) and self-reported SE (*r*(127) = -0.25, *p* = 0.005). Average self-reported TST was positively correlated with self-reported SE (*r*(146) = 0.44, *p* < 0.001).

Additionally, there was a significant negative association between DLMO phase and average self-reported TST (*r*(122) = − 0.27, *p* = 0.002) and between DLMO phase and MEQ (*r*(84) = − 0.33, *p* = 0.002), but DLMO phase was not significantly associated with any other primary sleep variable (i.e., TST, SE, and SP), all *p’s* > 0.05). MEQ was not associated with any other primary sleep variable (all *p’s* > 0.05).

### Sex differences in sleep, circadian, and activity measures

There were significant sex differences in sleep and circadian measures (see Table 1). Specifically, there were significant sex differences in actigraphy-measured number of SP (*F*(2, 126) = 5.84, *p* = 0.017, η^2^ = 0.043), such that females evidenced a greater number of SP, and in DLMO phase (*F*(2, 120) = 8.40, *p* = 0.004, η^2^ = 0.065), whereby males showed a later DLMO phase. There also were significant sex differences in self-reported SE (*F*(2, 143) = 4.18, *p* = 0.043, η^2^ = 0.028), whereby females reported less SE than males. No significant sex differences were found for actigraphy-measured TST (*F*(2, 126) = 1.78, *p* = 0.185, η^2^ = 0.013), actigraphy-measured SE (*F*(2, 126) = 0.23, *p* = 0.630, η^2^ = 0.001), MEQ score (*F*(2, 101) = 0.00, *p* = 0.971, η^2^ = 0.000), actigraphy-measured activity counts (*F(*2, 119) = 1.10, *p* = 0.30, η^2^ = 0.01), or DLMO-sleep onset time phase angle (*F*(2, 121) = 0.49, *p* = 0.49, η^2^ = 0.004). Similarly, no significant sex differences were found in self-reported TST (*F*(2, 145) = 0.00, *p* = 0.973, η^2^ = 0.000).

**Table 1:** Mean ± standard deviation (SD) of sex differences in sleep and circadian measures.

Measures	Females (*n* = 88)	Males (*n* = 62)	*F*	*p*	η^2^
Actigraphy total sleep time (min)	418.67 (50.72)	406.11 (56.86)	1.78	0.185	0.01
Actigraphy sleep efficiency (%)	83.65 (4.22)	83.30 (3.68)	0.23	0.630	0.00
Actigraphy sleep periods (#)	1.16 (0.16)	1.10 (0.14)	5.84*	0.017	0.04
DLMO (h)	21.72 (1.40)	22.46 (1.43)	8.40**	0.004	0.07
Activity counts (actigraphy-measured)	195.00 (53.00)	184.98 (46.06)	1.10	0.298	0.01
MEQ score	45.69 (8.36)	45.96 (9.75)	0.00	0.971	0.00
Self-report total sleep time (min)	437.03 (60.00)	437.72 (59.71)	0.00	0.973	0.00
Self-report sleep efficiency (%)	85.77 (9.43)	88.78 (7.42)	4.18*	0.043	0.03

*F* = *F*-statistic; *p* = *p*-value; η^2^ = eta squared; min = minutes; # = number; h = hour; *DLMO* Dim Light Melatonin Onset, *MEQ* Morningness-Eveningness Questionnaire.

*p ≤ 0.05; **p < 0.01.

### Racial differences in sleep, circadian, and activity measures

There were significant racial differences in all sleep measures, but not in the circadian measures (see Table 2). Specifically, there were significant racial differences in actigraphy-measured TST (*F*(2, 97) = 5.44, *p* = 0.022, η^2^ = 0.053), SE (*F*(2, 97) = 6.97, *p* = 0.010, η^2^ = 0.067), and number of SP (*F*(2, 97) = 5.87, *p* = 0.017, η^2^ = 0.053). White participants showed more actigraphy-measured TST and SE, as well as fewer total SP, compared to Black participants. There also were significant racial differences in the self-reported sleep indices. In line with the actigraphy-measured sleep findings, White participants evidenced more self-reported TST (*F*(2, 113) = 13.86, *p* < 0.001, η^2^ = 0.107) and better SE (*F*(2, 112) = 15.33, *p* < 0.001, η^2^ = 0.120). There were no significant racial differences in the circadian measures of DLMO phase (*F*(2, 94) = 0.22, *p* = 0.638, η^2^ = 0.002) or MEQ (*F*(2, 82) = 0.08, *p* = 0.779, η^2^ = 0.001), in actigraphy-measured activity counts (*F(*2, 92) = 0.59, *p* = 0.45, η^2^ = 0.01), or DLMO-sleep onset time phase angle (*F*(2, 95) = 0.89, *p* = 0.35, η^2^ = 0.01).

**Table 2:** Mean ± standard deviation (SD) of racial differences in sleep and circadian measures.

Measures	White (*n* = 84)	Black (*n* = 34)	*F*	*p*	η^2^
Actigraphy total sleep time (min)	419.77 (50.41)	391.55 (64.84)	5.44*	0.022	0.05
Actigraphy sleep efficiency (%)	84.11 (3.41)	81.98 (5.05)	6.97**	0.010	0.07
Actigraphy sleep periods (#)	1.11 (0.13)	1.20 (0.18)	5.87*	0.017	0.05
DLMO (h)	21.97 (1.47)	22.13 (1.67)	0.22	0.638	0.00
Activity counts (actigraphy-measured)	187.36 (53.23)	199.13 (43.20)	0.59	0.445	0.01
MEQ score	45.74 (9.60)	44.61 (7.24)	0.08	0.779	0.00
Self-report total sleep time (min)	451.08 (50.79)	404.53 (71.81)	13.86**	0.000	0.11
Self-report sleep efficiency (%)	89.09 (7.53)	82.39 (10.28)	15.33**	0.000	0.12

*F* = *F*-statistic; *p* = *p*-value; η^2^ = eta squared; min = minutes; # = number; h = hour; DLMO = Dim Light Melatonin Onset; MEQ = Morningness-Eveningness Questionnaire.

*p ≤ 0.05; **p < 0.01.

## Discussion

Our results show for the first time that sex and race both are significant predictors of several objective and self-reported sleep and circadian rhythm measures, controlling for BSD risk group differences, in a large sample of emerging adults participating in a naturalistic EMA study. Males exhibited later DLMO phase and fewer actigraphy-measured discrete sleep periods compared to females. Males also self-reported higher sleep efficiency than females. Actigraphy results indicated that White participants exhibited more total sleep time, better sleep efficiency, and fewer discrete sleep periods than Black participants. White participants also self-reported greater total sleep time and sleep efficiency compared to Black participants. Our study is the first to examine both objective and self-reported sleep and circadian parameters in a large, well-characterized, young adult sample. Furthermore, our findings represent a novel and meaningful contribution to the current literature, given that our measurement of sleep and circadian variables occurred in a naturalistic setting, allowing for external validity beyond what can be offered by laboratory studies. As such, the present findings address critical limitations of much of the previous literature (e.g., cross-sectional, mainly reliant on self-report, carried out in non-naturalistic laboratory settings), and thus inform ongoing research on sex and racial health disparities in sleep and in circadian rhythms.

With respect to actigraphy-measured sex differences in sleep, females in the current study exhibited more sleep periods than males, although their objective total sleep time and sleep efficiency did not differ. Our results contradict previous laboratory findings indicating that females objectively show longer sleep duration and better sleep efficiency than males^7,8,10^. It is possible that the measurement setting accounts for these differences, given that we measured sleep in a naturalistic setting, whereas previous studies were conducted in a laboratory. Perhaps females evince better sleep in the laboratory but encounter more environmental or social stressors in a naturalistic setting that prevent the same quality of sleep.

The current self-reported sex differences in sleep generally support previous findings indicating that males report fewer sleep problems and better sleep quality than females^12,13^. Our study results show that males self-reported better sleep efficiency, although their objective sleep efficiency did not differ. Inconsistencies between self-report and objective measures of sleep have been well documented in sleep research^10,38^. Given that females had more actigraphy-measured discrete sleep periods than males, it is possible that females perceived their sleep to be more fragmented, and thus poorer in quality, than did males.

Females in the current study also had earlier dim light melatonin onset times, indicating more phase advanced circadian rhythms, replicating earlier studies done in controlled lab environments^9,16^, and thus, generalizing these results to naturalistic settings, and thereby suggesting the sex difference in circadian rhythms is biological.

With respect to race, actigraphy findings demonstrated that Whites exhibited more total sleep time, better sleep efficiency, and fewer total sleep periods compared to Blacks. Self-reported racial differences in sleep fall in line with our objective measurements and with previous literature^17,25^; Whites in our study self-reported more sleep time and better sleep efficiency than Blacks. Our results support past findings demonstrating that Whites have better sleep efficiency than minority groups and corroborate existing evidence that Blacks sleep less overall, and exhibit less continuous sleep^17,23,25^. Despite evidence from previous research suggesting phase differences between Blacks and Whites^15,32–36^, there were no significant racial differences found for DLMO phase in the current study. Consistent with previous findings^32^, there were no significant racial differences for self-reported morningness-eveningness (chronotype).

Explanations for the observed racial disparities in sleep are likely complex. Given that some aspects of sleep have demonstrated heritability, genetic differences or other biological factors may exist that account for some racial differences in our findings^31,32^. However, it also is likely that a number of environmental and sociodemographic factors (e.g. stress, family burden, or neighborhood or economic effects) and health behaviors account for some racial differences in sleep duration and quality^30,32^. Given that sleep impacts many health outcomes (e.g., cognitive functioning, immune and hormone functioning, cardiovascular and mental health), future studies should strive to understand how genetic, environmental, sociodemographic, and mental health factors differentially contribute to racial differences and health disparities in sleep duration and quality.

The current study exhibits several notable strengths, including use of a well-characterized, representative, large emerging adult sample. Our young adult sample was demographically representative of a larger screened sample (N = 18,618) drawn from local high schools and universities in the Philadelphia, PA, USA region. These individuals were screened as part of the larger Teen Emotion and Motivation (TEAM) Project and selected based on high or moderate reward sensitivity. Indeed, there are benefits and drawbacks to conducting analyses with this specific sample. Noted benefits include the fact that our sample is demographically representative in terms of race and sex of young adults in a large metropolitan area, thus conferring high external validity to our findings, particularly compared to laboratory studies. Drawbacks include the fact that the sample was selected on the basis of high or low risk for developing BSD, thus limiting the generalizability of our findings to young adults more broadly. However, we controlled for BSD risk status in all analyses, removing the variance associated with BSD risk and decreasing the likelihoodthat BSD risk was a confound in our findings.

Our study also makes important contributions to understanding sleep and circadian function by utilizing both objective and self-report measures, allowing for a more accurate assessment of the constructs of interest in young adults’ daily lives. Employment of melatonin onset, actigraphy, and sleep diaries in a naturalistic environment allowed for a robust examination of sleep and circadian-related differences by sex and race. However, the study had some limitations. First, the analyses examining sleep differences by race were limited to White and Black participants, and behavioral reports were not used to constrain the sleep periods for the actigraphy data. Future research should examine other racial groups (e.g., Asian, American Indian, etc.) and add behavioral reports to corroborate the actigraphy data. In addition, future research should strive to replicate results with a view toward measuring latent variables, comparing objective and self-report sleep measurements more closely, and ultimately exploring race and sex-based differences in sleep and circadian characteristics in order to maximize the positive health outcomes associated with high quality sleep and circadian alignment.

## Methods

### Participants

Participants were recruited from the TEAM Project, a prospective study that identifies factors related to the onset and course of bipolar spectrum disorder (BSD)^39,40^. Participants were recruited from Philadelphia-area high schools and universities and participated in a two-phase screening process. In Phase 1, adolescent participants (*N* = 18,618) were administered two measures assessing behavioral activation system (BAS) sensitivity, with students scoring in the upper 15th percentile on both measures categorized as High BAS (HBAS), and students scoring between the 40th and 60th percentiles categorized as Moderate BAS (MBAS). Students who were included in the HBAS and MBAS (*n* = 1,180) groups were invited for Phase II screening, where they were administered self-report questionnaires and a diagnostic interview to assess mood and psychotic disorders. Participants were excluded from the study if they met *DSM-IV-TR* (American Psychiatric Association, 2000) criteria for a BSD or a psychotic disorder, or if they were not sufficiently fluent in English. The study was approved by the Institutional Review Board at Temple University, and all methods were performed in accordance with the relevant guidelines and regulations. All participants provided written informed consent before participating in the study. Only procedures and measures relevant to the current study are described here.

A subset of participants from Project TEAM falling into three groups^39,40^ were invited to complete the current study. Participants were 43 individuals with high behavioral activation system (BAS)/reward sensitivity and bipolar spectrum disorder (BSD), 64 individuals with high BAS/reward sensitivity and no history of BSD, and 43 individuals with moderate BAS/reward sensitivity and no history of BSD. Overall, 150 emerging adults (Mage = 21.8 years old, SD = 2.11; range = 18.6–27.9; 88 female; 84 White, 34 Black, 15 Asian or Pacific Islander, and 12 other ethnicity (e.g., Mexican, Native American, Hispanic) participated in the study.

### Measures

#### Objective sleep

To measure objective sleep parameters, participants wore an Actiwatch Spectrum (Philips Healthcare, Bend, OR), a well-validated motion-detecting device worn on the non-dominant wrist, continuously for 20 days, only removing it when it might get wet (e.g., bathing). Data were collected in 1-min epochs and scored using the Actiware software. Standard sleep parameters derived from actigraphy are moderately to strongly correlated with those derived from polysomnography in normal healthy sleepers^41^ and in clinical samples^42,43^. The following standard sleep parameters were derived from the actigraphy data: *total sleep time (TST)*, the number of minutes spent asleep between bedtime and final arising; *sleep efficiency (SE)*, the percentage of time spent asleep while in bed; and *sleep periods (SP)*, the number of discrete sleep periods within a day.

#### Self-reported sleep

To assess self-reported sleep parameters, participants were asked to answer questions about their sleep for 20 consecutive days on electronic sleep diaries. The sleep diary is reliable^44^ and is considered the gold standard for measuring self-reported sleep–wake rhythms^45^. The following standard sleep parameters were derived from the self-report diary data: TST and SE, as defined above.

#### Activity

Actigraphy provides an objective, reliable and valid method for assessing activity patterns with minimal restrictions on normal routines^46,47^. Participants wore an Actiwatch Spectrum (described above) for continuous measurement of activity level. Actiwatches recorded the number of times a participant moved their wrist; this has been shown to be a validated proxy for overall physical activity level^48^. We took a daily average of the number of times a participant moved their wrist (activity count) and averaged this over the 20-day study period.

#### Chronotype

The Morningness-Eveningness Questionnaire (MEQ)^37^ was administered to assess chronotype. It is a 19-item self-report well-validated measure of the morningness-eveningness dimension, with reliability coefficients ranging from 0.78 to 0.86 across samples in several countries^49–51^.

#### Dim light melatonin onset (DLMO)

Participants completed the DLMO procedure on 3 evenings during the EMA study: day 1, day 10, and day 20. Participants provided 10 saliva samples at 30-min intervals at home wearing light-attenuating goggles (Noir Medical Technologies, South Lyon, MI) beginning 5 h before their habitual bedtime. Participants were instructed to finish dinner at least 30 min before sampling began. No food was allowed during sampling and water was permitted only within 5 min after each sample. Nonsteroidal anti-inflammatory drugs (NSAID) were prohibited during the protocol, and alcohol and caffeine were prohibited for > 24 h before each DLMO procedure. Saliva (1.0–3.0 ml) was deposited into Salivette tubes using absorbent polyester swabs placed in the mouth for 5 min. Salivettes were refrigerated immediately and collected for continued storage at −20 °C in the laboratory pending assay. DLMO, a reliable marker of circadian phase^52,53^ was defined as the first interpolated point (derived from between two points) at 3.0 pg/ml on the rising curve of melatonin concentration. Melatonin was assayed with ELISA. The minimum detectable limit of the assay was 0.5 pg/ml with intra- and inter-assay coefficients of variation of 6.8% and 7.3%, respectively; 10% of samples were run in duplicate for quality control. DLMO-sleep onset time phase angle was calculated by subtracting DLMO onset clock time from sleep onset clock time.

### Statistical analysis

To ensure enough power to detect an effect, prior to collecting data for the study, power analyses were conducted with the G*Power 3.1 program^54^. We conducted a power analysis for ANCOVA with fixed effects. To detect significant main effects of sex and race, given Power (1 − β) = 0.80 and α = 0.05 to detect a medium effect, a total sample size of N = 90 is required. Thus, the analyses should be adequately powered to detect an effect.

Bivariate Pearson correlations (two-tailed, *p* < 0.05 significance level) were conducted between the primary sleep and circadian measures. We examined the associations between sex/race and group membership (moderate behavioral activation, high behavioral activation without BSD, and high behavioral activation with BSD). Pearson Chi-square Tests of Independence revealed that sex and group membership were not significantly associated (*χ*^2^(2) = 1.93, *p* = 0.38), whereas race and group membership were significantly related (*χ*^2^(2) = 6.82, *p* = 0.03). Thus, in all of our main analyses, we chose to control for group membership in order to mitigate the fact that there may be a pre-existing association between race and group membership. A series of ANCOVAs, controlling for initial group status, were conducted to evaluate differences on sleep, circadian, and activity characteristics by sex and race (*p* < 0.05 significance level). The actigraphy-measured and self-report sleep variables (TST, SP, and SE) were averaged over the 20-day study period, as were actigraphy-measured activity counts, which resulted in a single score for each measure. The DLMO was averaged over three time points (day 1, day 10, and day 20) to derive a single score. The MEQ was measured at baseline. For the analyses involving race, White participants were compared to Black participants. The sample sizes were too small to conduct comparisons among other races (e.g., Asian, Pacific Islander, American Indian/Alaskan Native).

## Data Availability

The dataset generated and analyzed during the current study is available from Dr. Lauren Alloy on a reasonable request.
